# Molecular dynamic simulation of performance of modified BAMO/AMMO copolymers and their effects on mechanical properties of energetic materials

**DOI:** 10.1038/s41598-020-75146-x

**Published:** 2020-10-23

**Authors:** Ke Wang, Huan Li, Jun-qiang Li, Hui-xiang Xu, Chao Zhang, Ying-ying Lu, Xue-zhong Fan, Wei-qiang Pang

**Affiliations:** Xi’an Modern Chemistry Research Institute, Xi’an 710065, China

**Keywords:** Theory and computation, Mechanical properties

## Abstract

Based on molecular dynamic method, densities, mechanical behavior and mechanical performance of P(BAMO/ AMMO) (Polymer 1) and two novel modified P(BAMO/AMMO) (Polymer 2: containing amino group, Polymer 3: containing nitro group), and their effects on mechanical properties of four energetic materials are investigated, the main results are as follow: Polymer 2 (1.235 g/cm^3^, 240 ± 5 K) and Polymer 3: 1.281 g/cm^3^, 181 ± 3 K) possess higher densities and lower glass transition temperatures than Polymer 1 (1.229 g/cm^3^, 247 ± 4 K). The modification makes Polymer 1 difficult to expand, improves its mechanical properties, but has few effect on its diffusion coefficient at same temperature and state. In addition, three binders are compatible with TNT, HMX and CL-20, and may react with DNTF. All polymers particularly improve rigidity of four energetic materials, and enhance their ductility except Polymer 2 on TNT. The ability of Polymer 2 and Polymer 3 improving rigidity (except Polymer 3 on HMX) and ductility of TNT and HMX is inferior to that of Polymer 1, but it is contrary for CL-20 and DNTF (except Polymer 2 on rigidity of DNTF). Moreover, Polymer 2-based interfacial crystals exhibit higher rigidity than Polymer 3-based interfacial crystals.

## Introduction

As with modern weapons continuous development, the requirements of their high energy, low sensitivity and low vulnerability are becoming more and more strict. Therefore, it is imperative to make modern weapons adapt to complicated and changeable situations in battlefield, especially their mechanical properties. The binders play an important role in the solid rocket propellants and polymer bonded explosives (PBXs), which are applied to splice components and endow mechanical properties of the systems^1–3^. However, traditional binders which possess low energy make it difficult to break through energy threshold of weaponry despite of their insensitivity. Therefore, it is urgent to design and prepare novel energetic and low-sensitive polymers, which can be used as binders in propellants and PBXs.

**Figure 1:**
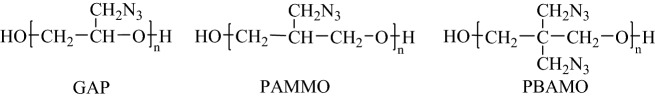
The structures of traditional azide energetic polymer binders.

Azide polymers (as shown in Fig. 1) started to be used as new energetic binders due to their high energy, high density, low sensitivity, and good mechanical properties^4–8^. The representative one is glycidyl azide polymer (GAP), which has been gradually applied to high-energy gun propellants, high-energy and low-signature propellants, and high-performance PBXs^9–11^. Otherwise, it is worth noting that 3,3′-bis(azidomethyl)oxetane polymer (PBAMO) is attracting attention of scientists. The density and heat of formation of PBAMO are higher than those of GAP, however, its mechanical property are weaker than that of GAP because of its higher glass transition temperature (PBAMO − 39 °C GAP − 45 °C)^12,13^. Therefore, it is necessary to modify PBAMO in order to make it satisfy application. Copolymerizing 3-azidomethyl-3-methyl oxetane (AMMO) into PBAMO is a feasible way to realize intramolecular plasticizing, because PAMMO is viscous liquid and has a similar structure with PBAMO^1,13^. Thus, the 3,3′-bis(azidomethyl)oxetane/3-azidomethyl-3-methyl oxetane copolymer (P(BAMO/AMMO)) began to be used as binder in solid rocket propellants^2,14–16^. Song et al.^16^ characterized the compatibility of P(BAMO/AMMO) with ordinary energetic materials in propellants, and results indicated that P(BAMO/AMMO) is compatible with cyclotrimethylene trinitramine (RDX), cyclotetramethylene tetranitramine (HMX), Al, hexanitrohexaazaiso-wurtzitane (CL-20) and ammonium perchlorate (AP), but medium reacting with 3,4-dinitrofurazanfuroxan(DNTF). Wang et al.^2^ prepared a propellant formation based on P(BAMO/AMMO) ETPE, which possessed highly theoretical specific impulse (2699.51 N s kg^-1^), low impact sensitivity (*H*_50_ = 45 cm) and steady combusting performance. Zhang et al.^17^ reported the response law of deflagration to detonation transition of P(BAMO/AMMO) based high energy propellants. Garaev et al.^15^ synthesized different mass ratio P(BAMO/AMMO) and found that while increasing fraction of PAMMO in the P(BAMO/AMMO) copolymers, the strength properties of the copolymers enhance and the degree of crystallinity decreases. However, there are few reports about modified P(BAMO/AMMO).

**Figure 2:**
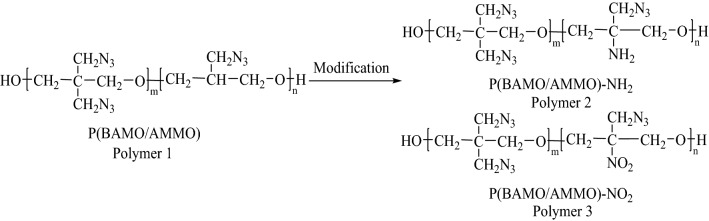
The structures of P(BAMO/AMMO) and modified P(BAMO/AMMO).

Because of expensive cost of experiments, molecular dynamic (MD) method is gradually applied to investigate performance of designed polymers, which can predict the glass transition temperature and mechanical properties of polymers, and analyze the influence of polymer on oxidizing agents^18,19^. Yang et al.^1^ studied the compatibility, intermolecular interactions, and mechanical properties of the various mass ratio P(BAMO/AMMO) and 1,5-diazido-3-nitrazapentane (DIANP) composites, which provides a practical guidance to the use of DIANP as a plasticizer for P(BAMO/AMMO). Lu et al.^20^ reported three designed modified GAP and compared their glass transition temperature and mechanical properties by MD method, and concluded that GAP-NO_2_ presented the most excellent comprehensive properties. Yang et al.^21^ studied the thermomechanical properties of an epoxy molding compound, and found simulated results are in good agreements with existing theoretical or experimentally measured values. Radhakrishnan et al.^22^ calculated performance parameters of designed energetic polymers derived from oxetanes with heterocyclic side chains with different energetic substituents, and results reveal that their performance are comparable to PAMMO and PBAMO. Therefore, the performance of modified BAMO/AMMO copolymers and their effects on properties of energetic materials can been predicted by MD method. The nitro group (–NO_2_) and amino group (–NH_2_) groups will be introduced to modify P(BAMO/AMMO) for enhancing the energy of P(BAMO/AMMO) and interaction between P(BAMO/AMMO) and energetic materials, respectively^20^, which may make P(BAMO/AMMO) better meet the requirement of modern weapons. The structures of P(BAMO/AMMO) and modified P(BAMO/AMMO) are presented in Fig. 2.

In this paper, the densities, glass transition temperatures, volumetric coefficient of thermal expansion, solution parameters and mechanical properties of P(BAMO/AMMO) and modified P(BAMO/AMMO) are predicted by MD method. Afterwards, the interfacial models of P(BAMO/AMMO) and modified P(BAMO/AMMO bonded with 2,4,6-trinitrotoluene (TNT), HMX, DNTF and CL-20 are constructed, and the effects of polymer binders on mechanical properties of TNT, HMX, DNTF and CL-20 are evaluated. The rest of paper is organized as follow: The details of construction of models and computational methods are presented in “Computational details" section. The results of properties of P(BAMO-AMMO) and modified P(BAMO-AMMO) and PBXs are discussed in "Results and discussions" section. In the end, the remarkable conclusions are summarized in "Conclusion".

## Computational details

### Choice of force field

A suitable force field is imperative to computational results, because the force field decides the degree of accuracy. In this paper, the COMPASS force field was used in MD calculation, which has been proved to be effective in investigating properties of condense phase^20,23–25^. All polymers and crystals were optimized by applying Forcite Module in Material Studio 8.0^26^. Meanwhile, Van der Waals force was calculated by atom-based method, and Ewald method was calculated by Electrostatic interaction^27,28^.

### Constructions of models and simulation methods for polymers

The amorphous cell of P(BAMO/AMMO) (m/n is 18/6) and modified P(BAMO/AMMO) were constructed by Amorphous Cell Module in Material Studio 8.0, and every cell contained 6 chains. The serial number of three polymer binders are respectively Polymer 1, Polymer 2 and Polymer 3 (as shown in Fig. 2). After structure optimization, the cell of P(BAMO/AMMO) and modified P(BAMO/AMMO) were relaxed by 50 ps constant particle number, pressure, and temperature (NPT) ensemble, 50 ps constant particle number, volume, and temperature (NVT) ensemble, and annealing simulation (temperature from 300 to 500 K). Then, the structure of minimum energy was used to perform NPT-MD simulation. The total calculated time was 1 ns, and the step time was 1 fs. The given temperature and pressure were 298 K and 100 kPa, respectively. Anderson method and Berendsen method were used to control temperature and pressure, respectively^29,30^. Initial velocity was sampled by Maxwell distribution, and velocity Verlet arithmetic was utilized^31^. Van der Waals force and Electrostatic interaction were calculated by atom-based method and Ewald method, respectively.

The balanced structure obtained from 1 ns NPT-MD simulation was repeatedly performed 500 ps NPT-MD simulation, where the temperature was successively set from 513 K to 33 K, and the interval of temperature was 20 K. After the simulation finished, the balanced structures were performed 100 ps NVT-MD simulation for every temperature. Then, every system in different temperatures was relaxed again. Ultimately, the relaxed structures in different temperatures were performed 1 ns NPT-MD simulation in their corresponding temperatures. The final results were applied to calculate volume (*V*), mean square displacement (*MSD*), non-bond energy (*NBE*) and mechanical properties.

### Constructions of models and simulation methods for PBXs

**Figure 3:**
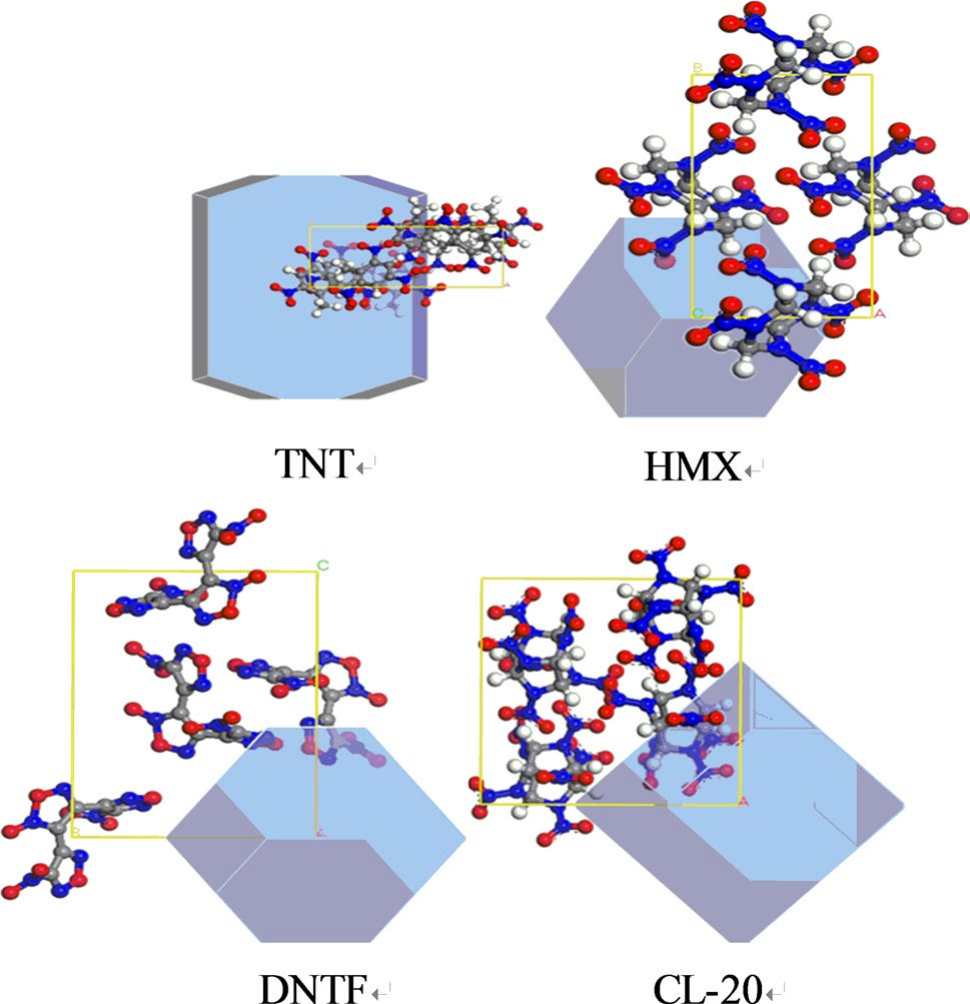
The morphology of four crystals in vacuum.

The Morphology Module was applied to evaluate the growth face of TNT, HMX, DNTF and CL-20 crystals in vacuum, and the original cell of all crystals are obtained from Cambridge Crystallographic Data Centre (CCDC). The growth faces of four crystals are presented in Fig. 3, and their first two growth faces and corresponding percentage are listed in Table 1.

**Table 1:** The first two growth faces and corresponding percentage of energetic materials.

Crystal	(*h k l*)	Percentage of total area (%)
TNT	(0 0 2)	38.6
	(2 0 0)	30.6
HMX	(0 1 1)	40.0
	(1 1 0)	31.4
DNTF	(0 1 1)	53.3
	(1 0 1)	24.4
CL-20	(0 1 1)	56.4
	(1 0 − 1)	18.6

**Figure 4:**
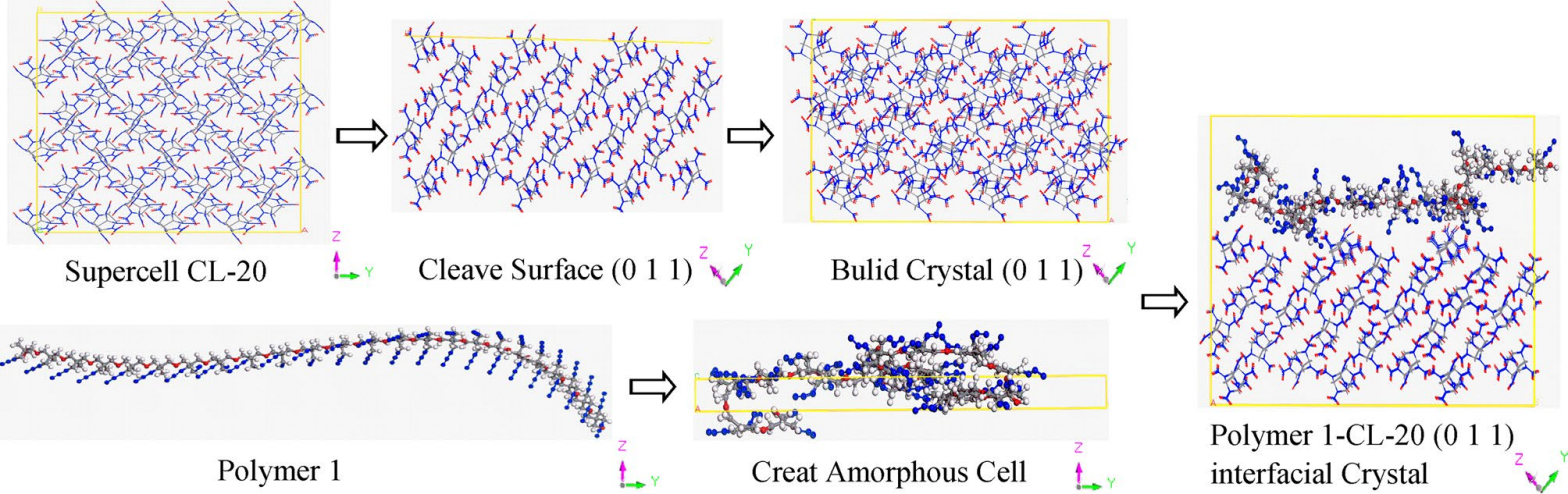
The process of creating interfacial crystal.

The construction of polymer bonded explosives models is as follow: taking CL-20 and Polymer 1 as an instance (Fig. 4): firstly, the 4*3*3 supercell of CL-20 was built, and was cleaved along the main growth face (0 1 1) ; secondly, the surface (0 1 1) was built as a crystal without vacuum; thirdly, the Polymer 1 chain was constructed as amorphous cell with same size as underside of crustal (0 1 1); finally, the crystal (0 1 1) and amorphous cell of Polymer 1 were built layer and their interfacial crystal was obtained. Repeating that method, the other interfacial crystals were acquired. It is worth noting that all steps were needed to be performed structure optimization to make energies of systems exhibit minimums after every step, because the system were deprived initial structure and possessed higher energy than its most stable structure after every step. As listed in Table 2, it is the number of total atoms of interfacial crystals and the mass ratio of polymer binder in every system. And the mass ratio of polymer binders in the systems was about 7.4%–8.4%.

**Table 2:** The number of total atoms of interfacial crystals and the mass ratio of polymer binder.

Systems	Number of Total atoms	Mass ratio of Polymer binder
Polymer 1/TNT(0 0 2)	4881	7.4%
Polymer 1/TNT(2 0 0)	4881	7.4%
Polymer 2/TNT(0 0 2)	4893	7.6%
Polymer 2/TNT(2 0 0)	4893	7.6%
Polymer 3/TNT(0 0 2)	4893	7.9%
Polymer 3/TNT(2 0 0)	4893	7.9%
Polymer 1/HMX(0 1 1)	4671	7.9%
Polymer 1/HMX(1 1 0)	4671	7.9%
Polymer 2/HMX(0 1 1)	4683	8.4%
Polymer 2/HMX(1 1 0)	4683	8.4%
Polymer 3/HMX(0 1 1)	4683	8.1%
Polymer 3/HMX(1 1 0)	4683	8.1%
Polymer 1/DNTF(0 0 1)	3881	7.3%
Polymer 1/DNTF(1 0 1)	3903	7.3%
Polymer 2/DNTF(0 0 1)	3893	7.5%
Polymer 2/DNTF(1 0 1)	3915	7.4%
Polymer 3/DNTF(0 0 1)	3893	7.8%
Polymer 3/DNTF(1 0 1)	3915	7.7%
Polymer 1/CL-20(0 1 1)	4359	7.5%
Polymer 1/CL-20(1 0 − 1)	4359	7.5%
Polymer 2/CL-20(0 1 1)	4371	7.6%
Polymer 2/CL-20(1 0 − 1)	4371	7.6%
Polymer 3/CL-20(0 1 1)	4371	7.9%
Polymer 2/CL-20(1 0 − 1)	4371	7.6%

The optimized structures of interfacial crystal and four supercell were performed 1 ns NPT-MD simulations, and the calculated method and set was same as chapter “2.2 Constructions of models and simulation methods for polymers”. The final 300 ps were used to predicted mechanical properties.

### Calculation of mechanical properties

**Scheme 1:**
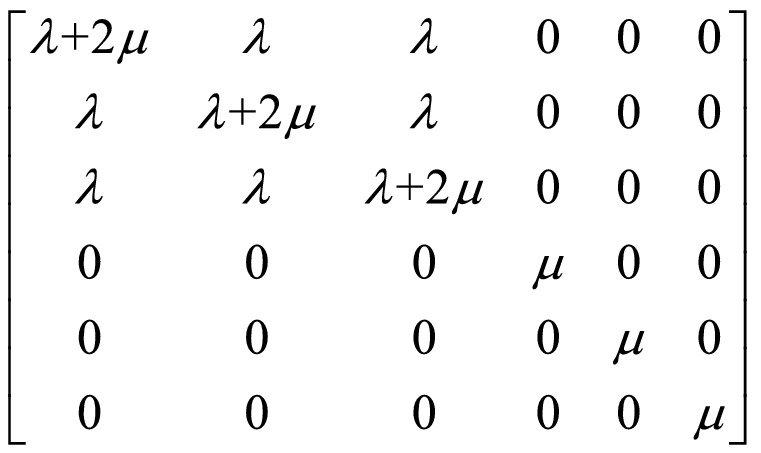
Stiffness matrix described by Lamé coefficients.

Based on the Hooke’s law and stiffness matrix for materials, the mechanical properties can be calculated. The generalized Hooke's law is often written as^32^:

1 $$\sigma_{i} = C_{ij} \varepsilon_{j}$$

where *σ*_*i*_ is the stress tensor(GPa), *ε*_*j*_ is the strain tensor(GPa), and *C*_*ij*_ is the 6 × 6 stiffness matrix of elastic constants. When the material is regarded as an isotropic material, the stiffness matrix of the stress–strain behavior can be fully expressed by Lamé coefficients (λ and μ), as follow Scheme 1.

Then, the Young's modulus (*E*, GPa), Bulk modulus (*K*, GPa), Shear modulus (*G*, GPa) and Poisson's ratio (*γ*) can be described by Lamé coefficients as follows^33–35^:

2 $$E = \mu \left( {\frac{3\lambda + 2\mu }{{\lambda + \mu }}} \right)$$

3 $$K = \lambda + \frac{2}{3}\mu$$

4 $$G = \mu$$

5 $$\gamma = \frac{\lambda }{{2\left( {\lambda + \mu } \right)}}$$

## Results and discussions

### Density and glass transition temperature of polymers

The densities of Polymer 1, Polymer 2 and Polymer 3 are 1.229 g/cm^3^, 1. 235 g/cm^3^ and 1.281 g/cm^3^, respectively. Therein, the density of Polymer 1 is close to its the reference value (1.25 g/cm^3^) and the relatively error is 1.7%, which indicates that the method applied in this work is reliable^14^. Otherwise, it is found that the –NH_2_ little improve the density of Polymer 1, and the –NO_2_ well increase the of Polymer 1.

**Figure 5:**
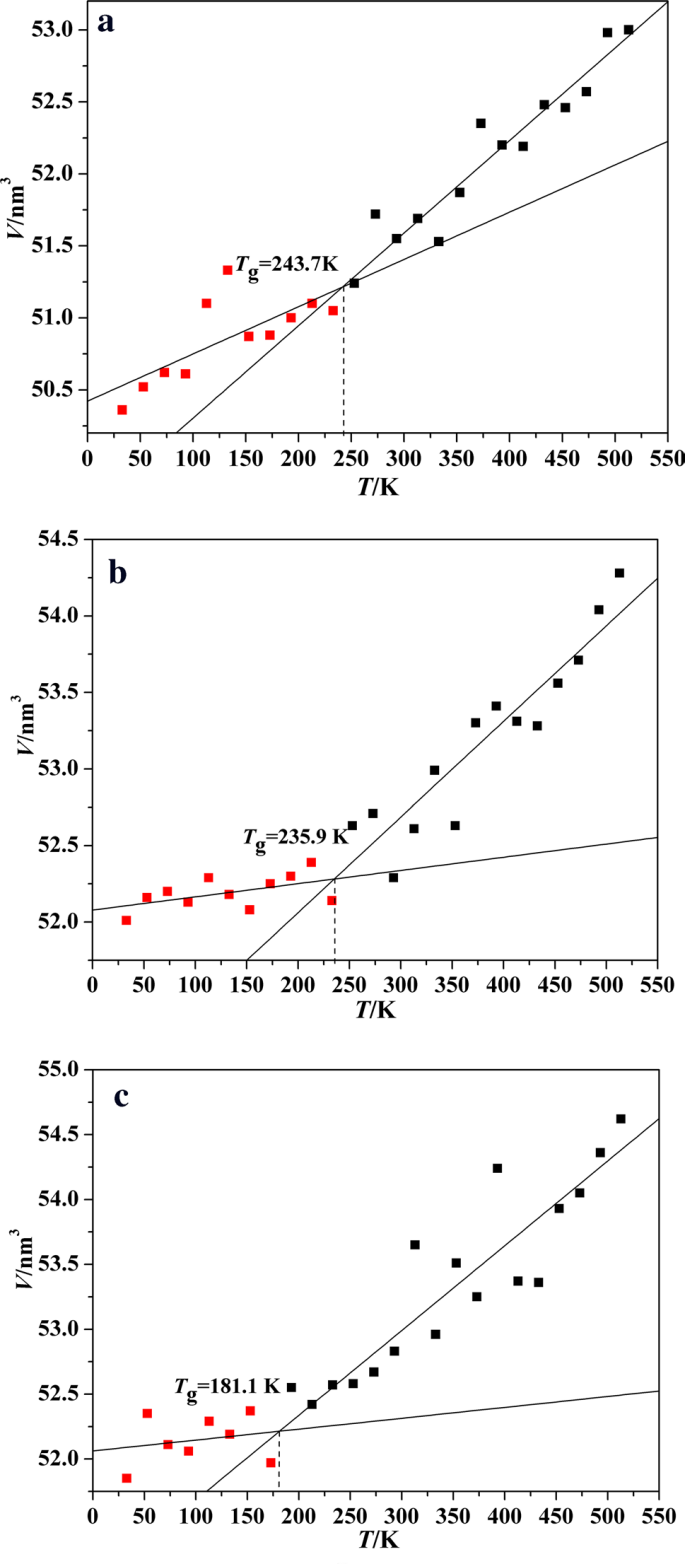
The volume vs temperature curves of P(BAMO/AMMO) and modified P(BAMO/AMMO).

The glass transition temperature (*T*_g_) is probable one of the most important properties of polymers because it determines the processing and working temperature range. A large number of methods have been applied to evaluate the *T*_g_ of polymer based on molecular dynamic method^20,21^. For each temperature, the volume of the polymer model was obtained from the whole duration of MD simulation and averaged. It is worth noting that every 1 ns NPT-MD simulation in different temperature is performed based on 500 ps NPT-MD and 100 ps NVT-MD relaxation in corresponding temperature, therefore, the errors of averaged volume at each temperature can be negligible. The volume of three polymers at different temperature is listed in Table 3, and the curves of their volume versus temperature are presented in Fig. 5. It is easy to see, there are obvious discontinuities in the slope of the curves, which means the polymers exist the glass transition, namely, the polymer transforms from rubbery state to glassy state. The *T*_g_ is predicted by performing the segmental linear regression of the data. Eventually, the *T*_g_ of Polymer 1 Polymer 2 and Polymer 3 are respectively 243.7 K, 235.9 K and 181.1 K. The *T*_g_ of Polymer 1 is close to its literature value (244.7 K), and the relative error is 0.4%, which also shows that this work is reliable^16^. Meanwhile, it is obvious that the introduced functional groups all make the *T*_g_ of P(BAMO-AMMO) decrease, and nitro group particularly reduces the *T*_g_.

**Table 3:** The volume of P(BAMO/AMMO) and modified P(BAMO/AMMO) at different temperature.

T/K	*V*/nm^3^		
	Polymer 1	Polymer 2	Polymer 3
513	53.00	54.28	54.62
493	52.98	54.04	54.36
473	52.57	53.71	54.05
453	52.46	53.56	53.93
433	52.48	53.28	53.36
413	52.19	53.31	53.37
393	52.20	53.41	54.24
373	52.35	53.30	53.25
353	51.87	52.63	53.51
333	51.53	52.99	52.96
313	51.69	52.61	53.65
293	51.55	52.19	52.83
273	51.72	52.71	52.67
253	51.24	52.63	52.58
233	51.05	52.14	52.57
213	51.10	52.39	52.42
193	51.00	52.30	52.55
173	50.88	52.25	51.97
153	50.87	52.08	52.37
133	51.33	52.18	52.19
113	51.10	52.29	52.29
93	50.61	52.13	52.06
73	50.62	52.20	52.11
53	50.52	52.16	52.35
33	50.36	52.01	51.85

Otherwise, the volumetric coefficient of thermal expansion (*α)* of three polymer binders can be also evaluated by the curves of volume vs temperature, and the *α* is defined by^36^:

5 $$\alpha = \frac{1}{{V_{{0}} }}\left( {\frac{\partial V}{{\partial T}}} \right)$$

where *V*_0_ is the original volume of equilibrium system before cooling.

The (*∂V/∂T*) values of three systems in rubbery state and glassy state and *α* values in three temperatures are shown in Table 4. When the polymers exhibit rubbery state in same temperature, it is easy to find the *α* values of Polymer 3 and Polymer 2 are close to each other and are lower than that of Polymer 1. It indicates that Polymer 1 is easiest to expand at room and high temperature, which may be easy to introduce damage of the propellants or PBXs systems. In glassy state, the Polymer 1 is easiest to expand and Polymer 2 is much difficult to expand. It means that Polymer 2 and Polymer 3 are more difficult to expand than Polymer 1 in the same state and temperature. Otherwise, when temperature is 181.1 K < T < 235.9 K, Polymer 3 significantly expand than Polymer 1 and Polymer 2, when temperature is 235.9 K < T < 243.7 K, Polymer 3 and Polymer 2 particularly expand than Polymer 1.

**Table 4:** The *D* and related parameters of thermal expansion of P(BAMO/AMMO) and modified P(BAMO/AMMO).

System	(*∂V/∂T*) × 10^3^/nm^3^/K		*α* × 10^4^*/K*^-1^	
	Glassy state	Rubbery state	Glassy state	Rubbery state
Polymer 1	3.28	6.43	0.64–0.65	1.21–1.24
Polymer 2	0.86	6.25	0.16–0.17	1.15–1.20
Polymer 3	1.92	6.33	0.37	1.16–1.21

**Figure 6:**
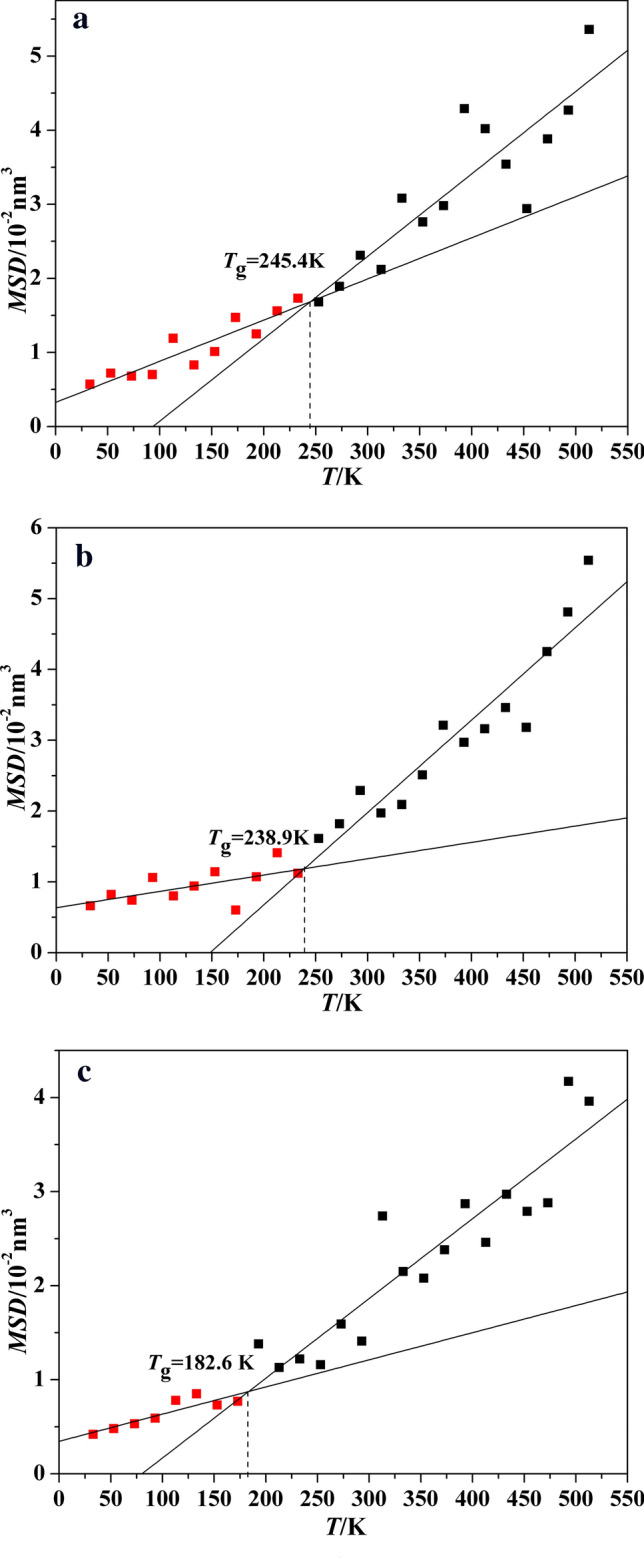
The *MSD* vs temperature curves of PBAMO-AMMO and modified PBAMO-AMMO.

Because the polymers continue to move in system and its movement is constrained by space and temperature, the *T*_g_ can also be predicted by *MSD* vs temperature curve. As is shown in Fig. 6, there are the *MSD* vs temperature curves of P(BAMO/AMMO) and modified P(BAMO/AMMO). Meanwhile, the specific values of their *MSD* are listed in Table 5. Ultimately, the *T*_g_ values of Polymer 1, Polymer 2 and Polymer 3 evaluated by the fitted curves are 245.4 K, 238.9 K and 182.6 K, respectively. The acquired variation trend is agreed with that obtained by volume-temperature data, namely, nitro group significantly reduces the *T*_g_ of P(BAMO/AMMO). In addition, it is found that the *T*_g_ values of three polymers obtained by *MSD*-temperature data are all slightly higher than those acquired by volume-temperature data.

**Table 5:** The *MSD* of P(BAMO/AMMO) and modified P(BAMO/AMMO) at different temperature.

T/K	*MSD*/10^–2^ nm^2^		
	Polymer 1	Polymer 2	Polymer 3
513	5.36	5.54	3.96
493	4.27	4.81	4.17
473	3.88	4.25	2.88
453	2.94	3.18	2.79
433	3.54	3.46	2.97
413	4.02	3.16	2.46
393	4.29	2.97	2.87
373	2.98	3.21	2.38
353	2.76	2.51	2.08
333	3.08	2.09	2.15
313	2.12	1.97	2.74
293	2.31	2.29	1.41
273	1.89	1.82	1.59
253	1.68	1.61	1.16
233	1.73	1.12	1.22
213	1.56	1.41	1.13
193	1.25	1.07	1.38
173	1.47	0.60	0.77
153	1.01	1.14	0.73
133	0.83	0.94	0.85
113	1.19	0.80	0.78
93	0.70	1.06	0.59
73	0.68	0.74	0.53
53	0.72	0.82	0.48
33	0.57	0.66	0.42

The migration of plasticizer is related to its diffusion coefficient (*D*), which depends on the *MSD* and time (*t*) of MD simulation. Therefore, *D* is described in Eq. (6) based on the relation of Einstein^37,38^:

6 $$D = \mathop {\lim }\limits_{t \to \infty } \frac{{\left\langle {\left| {r(t) - r(0)} \right|^{2} } \right\rangle }}{6t}$$

where *r*(*t*) is coordinate of plasticize in *t*, and *r*(0) is the original coordinate.

The relationship of *MSD* is calculated by Eq. (7)^38^:

7 $$MSD = s(t) = \left\langle {\left| {r(t) - r(0)} \right|^{2} } \right\rangle$$

The *D* is ultimately estimated by Eqs. (6) and (7)^38^:

8 $$D = s(t)/6t = m/6$$

**Figure 7:**
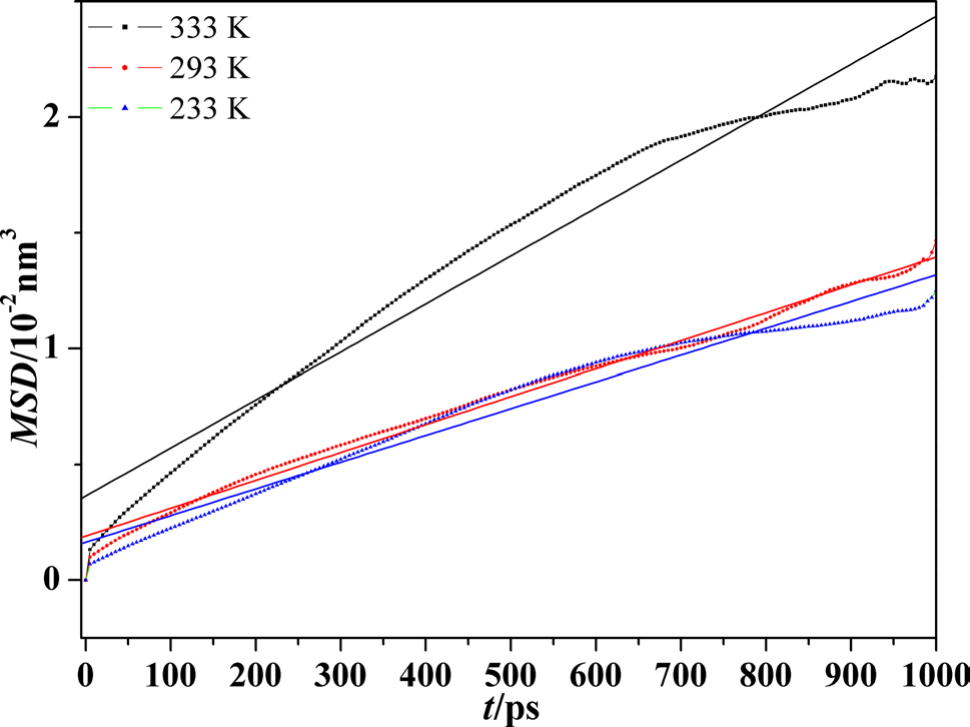
The *MSD*-t and their fitting curves of Polymer 3 at three temperatures.

The migration of three polymers almost in low temperature (233 K), room temperature (293 K) and high temperature (333 K) are compared. The Fig. 7 plots the *MSD*-t curves and their fitting curves of Polymer 3 in the above three temperature. The *D* and *m* values of three polymers are contrasted in Table 6. It is worth noting that the *D* values (0.19 × 10^-11^m^2^/s-0.35 × 10^-11^m^2^/s) of three polymers at three temperature approximate to each other due to their long molecular chains, which means introduced groups have few influence on the migration of Polymer 1. Therein, Polymer 3 is much difficult to migrate at 233 K despite it is rubbery state and others are glassy state.

**Table 6:** The *MSD* of P(BAMO/AMMO) and modified P(BAMO/AMMO) at different temperature.

T/K	*m*	*D*	*m*	*D*	*m*	*D*
	× 10^11^m^2^/s					
	233 K		293 K		333 K	
Polymer 1	1.33	0.22	2.05	0.34	1.66	0.28
Polymer 2	1.45	0.24	1.88	0.31	1.99	0.33
Polymer 3	1.15	0.19	1.20	0.20	2.07	0.35

**Figure 8:**
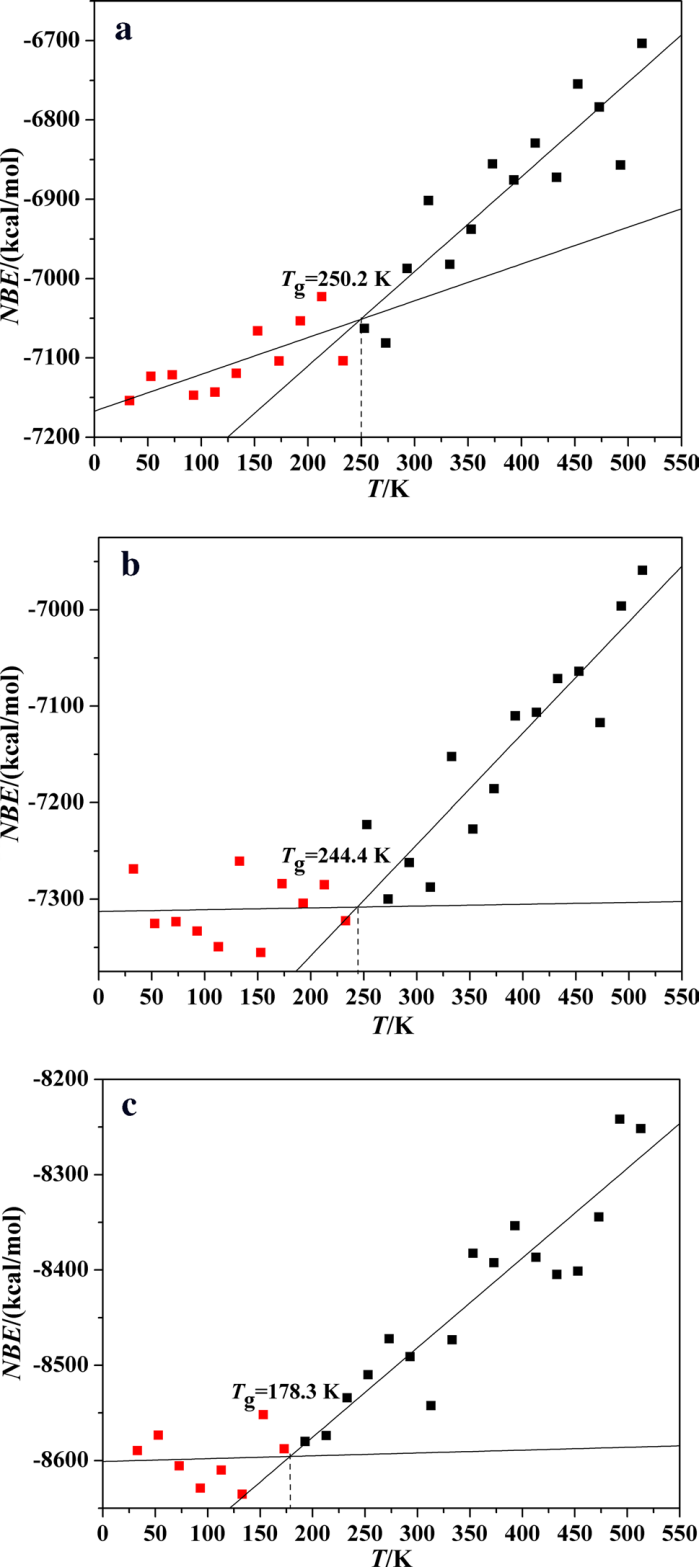
The *NBE* vs temperature curves of P(BAMO/AMMO) and modified P(BAMO/AMMO).

Non-bond energy (*NBE*) also possesses a discontinuity near the glass transition temperature^21^. Therefore, the *T*_g_ can be estimated by fitting *NBE*-temperature curves (as shown in Fig. 8). And the related data are presented in Table 7. By fitting the curves, the *T*_g_ values of Polymer 1, Polymer 2 and Polymer 3 are 250.2 K, 244.4 K and 178.3 K, respectively. And the variation trend of three polymers is according to the former. Meanwhile, it can be discovered that the *T*_g_ values of Polymer 1 and Polymer 2 obtained by *NBE*-temperature data are all slightly higher than those acquired by volume-temperature and *MSD*-temperature data, however, Polymer 3 is contrary. It may result from deviation.

**Table 7:** The *NBE* of P(BAMO/AMMO) and modified P(BAMO/AMMO) at different temperature.

T/K	*NBE*/ kcal/mol		
	Polymer 1	Polymer 2	Polymer 3
513	− 6703.639	− 6959.134	− 8251.764
493	− 6856.954	− 6996.295	− 8241.802
473	− 6783.718	− 7117.207	− 8344.430
453	− 6754.718	− 7063.942	− 8401.369
433	− 6872.562	− 7071.587	− 8404.741
413	− 6829.307	− 7106.599	− 8386.747
393	− 6875.732	− 7110.142	− 8353.722
373	− 6855.513	− 7185.726	− 8392.551
353	− 6937.900	− 7227.344	− 8382.516
333	− 6982.180	− 7152.461	− 8473.233
313	− 6901.687	− 7287.527	− 8542.490
293	− 6987.371	− 7262.041	− 8491.098
273	− 7081.211	− 7299.944	− 8472.283
253	− 7062.839	− 7222.597	− 8509.974
233	− 7103.762	− 7322.598	− 8534.174
213	− 7022.837	− 7285.102	− 8573.779
193	− 7053.523	− 7304.441	− 8579.951
173	− 7104.002	− 7284.087	− 8587.683
153	− 7066.050	− 7355.428	− 8551.878
133	− 7119.629	− 7260.640	− 8635.377
113	− 7143.169	− 7349.464	− 8610.065
93	− 7147.256	− 7333.155	− 8629.005
73	− 7121.449	− 7323.479	− 8605.537
53	− 7123.356	− 7325.335	− 8573.377
33	− 7153.965	− 7268.767	− 8589.562

In a conclusion, based on calculating *V*, *MSD* and *NBE*, the *T*_g_ values of three polymer are respectively 247 ± 4 K, 240 ± 5 K and 181 ± 3 K by assessing the range of every kind of *T*_g_. The -NH_2_ and -NO_2_ group all decrease the glass transition temperature of P(BAMO/AMMO), and the reducing effect of -NO_2_ group is better.

### Mechanical properties of polymer and its miscibility with energetic materials

Mechanical properties are mainly due to the behavior exhibited by polymeric systems under different testing modes of elastics properties. Binders play an important role in the solid rocket propellants and PBXs, whose mechanical properties have significant effects on the mechanical properties of systems. The mechanical properties of P(BAMO/AMMO) and modified P(BAMO/AMMO) are listed in Table 8. It is easy seeing that the *E* and G values of Polymer 1 have a little diminution when introducing -NH_2_ and -NO_2_ group, however, its *K* value slightly increase. Therefore, there is a conclusion that the Polymer 2 and Polymer 3 become more plastic than Polymer 1, namely, the former is more effortless to deformed, which may make the propellants or PBXs systems easier to process. Ratio of *K*/*G* can be also used to evaluate the ductility of materials and with higher value expected. Therefore, the Polymer 2 and Polymer 3 possess better ductility than the Polymer 1 by comparing their *K*/*G* values.

**Table 8:** The mechanical properties of P(BAMO/AMMO) and modified P(BAMO/AMMO).

	Polymer 1	Polymer 2	Polymer 3
C_11_	5.798	6.039	5.659
C_12_	1.798	1.448	1.625
C_13_	1.934	1.557	1.700
C_22_	5.384	5.619	5.478
C_23_	2.096	1.746	1.760
C_33_	5.564	4.348	5.750
C_44_	1.481	2.092	1.129
C_55_	2.044	1.490	1.397
C_66_	1.799	0.886	1.515
λ	2.033	2.357	2.935
μ	1.775	1.489	1.347
*E*/GPa	4.50	3.89	3.61
*K*/GPa	3.22	3.35	3.83
*G*/GPa	1.77	1.49	1.35
*γ*	0.27	0.31	0.34
*K*/*G*	1.82	2.25	2.84

Miscibility means whether the polymer and energetic materials can mix in all proportions and ultimately form a homogeneous state. And cohesive energy density and solubility parameter are important index to estimate the miscibility of two components, which can be calculated by MD methods. If the solubility parameters of two components are more close, their compatibility are better, namely, they are miscible. As presented in Table 9, there are the cohesive energy density and solubility parameter of P(BAMO/AMMO), modified P(BAMO/AMMO) and four energetic materials. The cohesive energy density and solubility parameters of three polymer are close to each other despite of introducing functional groups, which indicates the functional groups have little influence on the solubility parameter of P(BAMO/AMMO). Otherwise, the solubility parameters of four energetic materials and three binders are 28.00–31.70 (J/cm^3^)^1/2^ and 19.29–19.92 (J/cm^3^)^1/2^, respectively. It is obtained the number of their difference value (|Δ*δ*|) is 8.08 (J/cm^3^)^1/2^ <|Δ*δ*|< 12.41 (J/cm^3^)^1/2^. Therein, the |Δ*δ*| of DNTF is largest than the other when corresponding to same polymer. Otherwise, previous reports^16^, as listed in Table 10, proved that Polymer 1 was compatible with HMX and CL-20, but medium reacted with DNTF. Therefore, it can be concluded that Polymer 2 and Polymer 3 are compatible with TNT, HMX and CL-20, but may be incompatible with DNTF as same as Polymer 1.

**Table 9:** Cohesive energy density and Solubility parameter of P(BAMO/AMMO), modified P(BAMO/AMMO) and energetic materials.

Component	CED	Solubility parameter
	J/cm^3^	(J/cm^3^)^1/2^
TNT	784	28.00
HMX	959	30.97
DNTF	1005	31.70
CL-20	927	30.45
Polymer 1	370	19.29
Polymer 2	397	19.91
Polymer 3	397	19.92

**Table 10:** Compatibility of P(BAMO/AMMO) with some energetic materials^16^.

Number	Component	Outgassing amount/mL	Net outgassing amount/mL	Evaluation
0	P(BAMO/AMMO)	0.46		
1	HMX	0.09	− 0.19	Compatible
	P(BAMO/AMMO)/HMX	0.36		
2	CL-20	0.25	2.40	Compatible
	P(BAMO/AMMO)/CL-20	3.11		
3	DNTF	0.82	4.02	Medium reacting
	P(BAMO/AMMO)/DNTF	5.30		

### Mechanical properties of different PBXs

**Figure 9:**
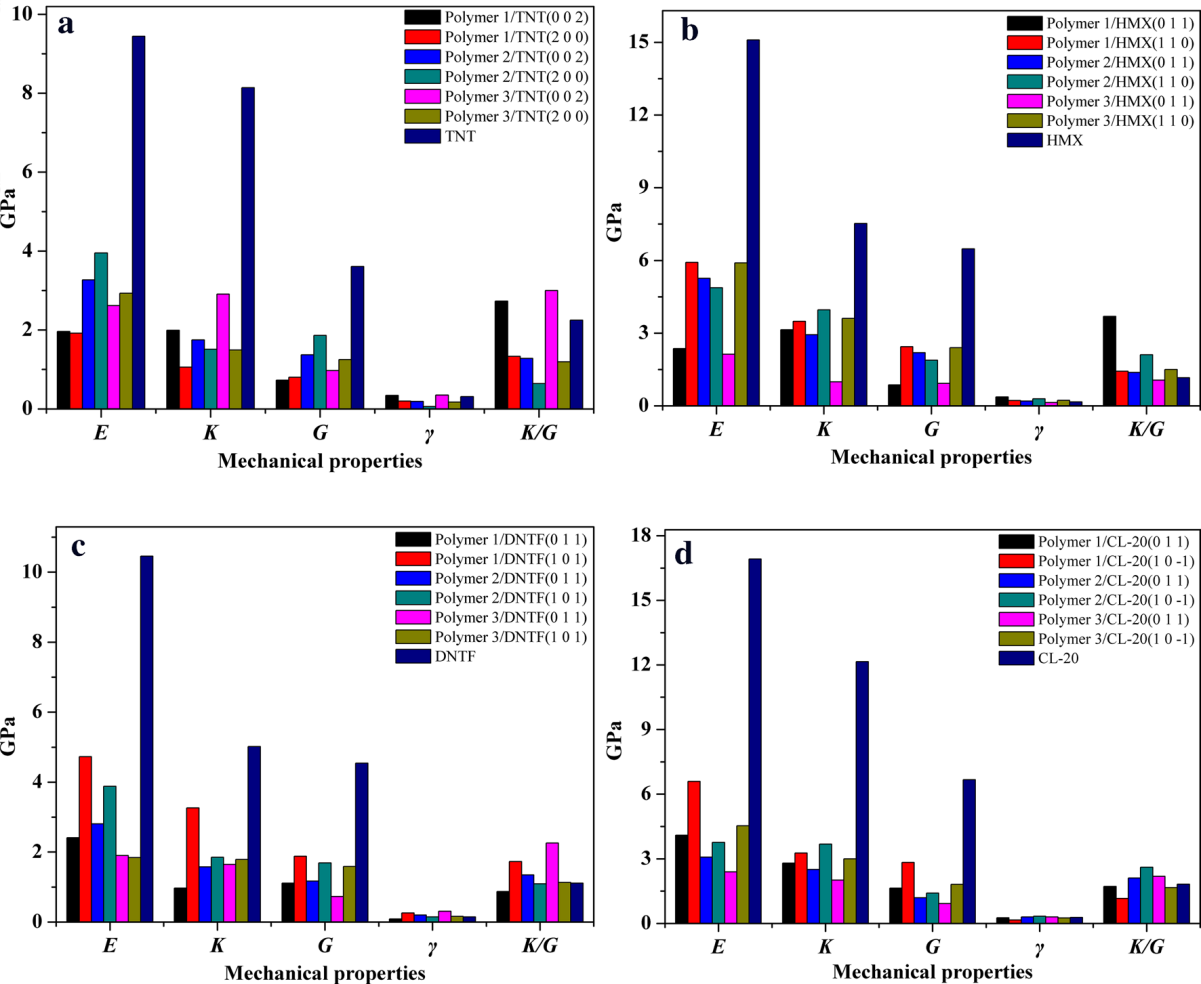
The mechanical modulus of different polymer binder/energetic materials interfacial crystals. Note: The *γ* and *K*/*G* values are dimensionless.

Energetic materials used as oxidation are important solid components in solid rocket propellant and polymer bonded explosives, whose mechanical properties significantly depend on energetic materials. Meanwhile, binders which are applied to bond solid components also play an important role in improving the mechanical properties of the propellants of PBXs system. The mechanical parameters of different energetic material/binder systems calculated by MD method are presented in Table 11–14. Moreover, their mechanical modulus are plotted in Fig. 9.

**Table 11:** The mechanical parameters of polymer/TNT interfacial crystal.

System	Polymer 1/TNT		Polymer 2/TNT		Polymer 3/TNT		TNT
	(0 0 2)	(2 0 0)	(0 0 2)	(2 0 0)	(0 0 2)	(2 0 0)	
C_11_	3.3123	1.2512	4.4243	3.5902	4.5253	3.2174	9.1342
C_12_	1.4507	0.3503	1.8329	2.4855	2.1455	0.7998	4.1709
C_13_	1.1450	0.3554	1.4024	2.4481	1.7067	1.6574	7.7870
C_22_	4.4368	2.4138	3.0556	4.6209	4.7446	1.6375	16.6441
C_33_	1.1597	2.7162	3.2727	3.7412	3.3299	4.6307	13.1045
C_44_	0.8161	0.9648	1.3908	2.3663	0.5955	0.3149	2.2549
C_55_	0.7745	0.7121	1.4369	1.7239	1.6991	2.1585	5.4733
C_66_	0.6130	0.7256	1.2957	1.4832	0.6140	1.2890	3.1115
λ	1.5005	0.5254	0.8353	0.2685	2.2608	0.6536	5.7345
μ	0.7345	0.8008	1.3745	1.8578	0.9695	1.2541	3.6132
*E*/GPa	1.96	1.92	3.27	3.95	2.62	2.93	9.44
*K*/GPa	1.99	1.06	1.75	1.51	2.91	1.49	8.14
*G*/GPa	0.73	0.80	1.37	1.86	0.97	1.25	3.61
*γ*	0.34	0.20	0.19	0.06	0.35	0.17	0.31
*K*/*G*	2.73	1.33	1.28	0.64	3.00	1.19	2.25

#### Binder/TNT interfacial crystals

The percentage of (0 0 2) in total growth face of TNT is regarded as 38.6% in this paper, which consists of (0 0 2) and its symmetry plane (0 0 − 2) possessing same proportion. The (2 0 0) is in the same case with (0 0 2). Therefore, (0 0 2) and (2 0 0) are regarded as the first two growth faces of TNT. As listed in Table 11, there are mechanical parameters of polymer binder/TNT interfacial crystals and pure TNT. It can be found that the mechanical modulus of all interfacial crystals are significantly lower than those of pure TNT, which suggests that three polymer binders can well decrease the rigidity of TNT. Meanwhile, when the binder is Polymer 1, the Young’s modulus (*E*), bulk modulus (*K*) and shear modulus (*G*) values of corresponding interfacial crystals are smallest in spite of they obtained from TNT (0 0 2) or TNT (2 0 0) (except the *K* value of Polymer 1/TNT (0 0 2) crystal), which indicates that modified P(BAMO/AMMO) do not well enhance the ductility of TNT compared with P(BAMO/ AMMO). Otherwise, the mechanical modulus of Polymer 3/TNT crystal are all lower to those of Polymer 2/TNT crystal for the same interface (except the *K* value of Polymer 3/TNT(0 0 2) crystal ). Therefore, the extent of polymer binders reducing the rigidity of TNT decease in the order: Polymer 1 > Polymer 3 > Polymer 2. However, only the *K*/*G* and *γ* values of Polymer 1/TNT(0 0 2) and Polymer 3/TNT(0 0 2) surpass those of TNT, and the *K*/*G* and *γ* values of Polymer 2/TNT interfacial crystal are all lower than those of TNT, which means that only Polymer 1 and Polymer 3 partly improve the ductility of TNT and Polymer 2 fails. In a conclusion, the order of the ability of polymer binders improving ductility of TNT is: Polymer 3≈ Polymer 1 > Polymer 2.

#### Binder/HMX interfacial crystals

Compared with pure HMX crystal, the mechanical modulus of binder/HMX interfacial crystals all significantly reduce, which indicates that binders all effectively improve the isotropy of HMX crystal (as shown in Table 12). The *E*, *K* and *G* values of polymer binder/HMX (0 1 1) (the first growth face) crystal are particularly lower than those of polymer binder/HMX (1 1 0) (the second growth face) crystal when binder are Polymer 1 and Polymer 3. However, while binder is Polymer 2, the mechanical modulus of the *E* and *G* values of polymer binder/HMX (0 1 1) crystal are even little larger than those of binder/HMX (1 1 0) crystal, which notes the effects of Polymer 2 on the two main growth faces of HMX have no obvious distinction. Otherwise, as presented in Fig. 8b, by comparing the elastic constants of binder/HMX interfacial crystal, when interface is (0 1 1) face of HMX crystal, the binders increase the plasticity of interfacial crystals in the order as follow: Polymer 3 > Polymer 1 > Polymer 2. Nevertheless, while being (1 1 0) face, the order is Polymer 2 > Polymer 3≈ Polymer 1. Overall, considering the percentage of main growth faces and the number of elastic constants of interfacial crystals, the ability of binder reducing the rigidity of HMX decease in the order: Polymer 3 > Polymer 1 > Polymer 2. Otherwise, except Polymer 3/ HMX (0 1 1) interfacial crystal, the *K*/*G* and *γ* values of the other all exceed those of HMX. Therein, those of Polymer 1/HMX (0 1 1) and Polymer 2/HMX (1 1 0) are the largest one and second one, respectively. Therefore, Polymer 1 and Polymer 2 can improve the ductility of HMX crystal, respectively. Their extent of improving ductility of HMX decreases in the order: Polymer 1 > Polymer 2 > Polymer 3.

**Table 12:** The mechanical parameters of polymer/HMX interfacial crystal.

System	Polymer 1/HMX		Polymer 2/HMX		Polymer 3/HMX		HMX
	(0 1 1)	(1 1 0 )	(0 1 1)	(1 1 0 )	(0 1 1)	(1 1 0 )	
C_11_	7.7586	5.3423	6.7112	4.6590	5.5092	14.1389	19.5187
C_12_	1.4438	2.8568	1.3083	2.0664	1.5760	0.2865	6.5903
C_13_	1.0238	2.1558	2.8761	1.7282	1.0367	1.7432	6.1328
C_22_	2.4474	7.4991	3.4972	6.8800	0.6030	1.2756	15.2861
C_33_	2.6393	7.3024	7.3544	7.8582	0.5876	5.0232	16.6834
C_44_	0.4772	2.0291	1.0529	2.0449	1.4279	2.5897	6.3866
C_55_	1.2996	2.1180	2.6247	1.6059	0.6171	2.3234	7.0231
C_66_	0.8020	3.1656	2.8991	1.9906	0.7466	2.2920	11.8656
λ	2.5625	1.8394	1.4698	2.7048	0.3723	2.0091	3.1960
μ	0.8596	2.4376	2.1923	1.8805	0.9305	2.4017	6.4815
*E*/GPa	2.36	5.92	5.26	4.87	2.13	5.90	15.10
*K*/GPa	3.14	3.49	2.93	3.96	0.99	3.61	7.52
*G*/GPa	0.86	2.44	2.19	1.88	0.93	2.40	6.48
*γ*	0.37	0.22	0.20	0.29	0.14	0.23	0.16
*K/G*	3.69	1.43	1.38	2.11	1.06	1.50	1.16

#### Binder/DNTF interfacial crystals

The data of mechanical parameters of binder/DNTF interfacial crystals and pure DNTF are listed in Table 13. It is easy to see that three polymer binders also effectively enhance the isotropy of DNTF crystal. As the first growth face, the mechanical modulus of interfacial crystals corresponding to DNTF (0 1 1) face are lower than those corresponding to DNTF (1 0 1) face (except the *E* values of Polymer 3/DNTF interfacial crystals), which will contribute to improving the mechanical properties of DNTF. Otherwise, it is worthy noting that the *E*, *K* and *G* values of Polymer 3/DNTF interfacial crystals are all lower than the other when corresponding to same growth face (except the *K* value of the Polymer 3/DNTF (0 1 1) are little larger than the other). Moreover, the *E*, *K* and *G* values of Polymer 2/DNTF (0 1 1) interfacial crystal are all larger than those of Polymer 1/DNTF (0 1 1) interfacial crystal, which is corresponding to the first growth face of DNTF (53.3%). However, the *E*, *K* and *G* values of Polymer 2/DNTF (1 0 1) interfacial crystal are all lower than those of Polymer 1/DNTF (1 0 1) interfacial crystal, which is corresponding to the second growth face of DNTF (24.4%). The comparison of their mechanical parameters is presented in Fig. 8c, and there is a conclusion that the extent of polymer binders improving the rigidity of DNTF deceases in the order: Polymer 3 > Polymer 1 > Polymer 2. Otherwise, the *K*/*G* and *γ* values of Polymer 1/DNTF (1 0 1), Polymer 2/DNTF(0 1 1) and Polymer 3/DNTF interfacial crystals are all superior to those of DNTF crystal. Therein, those of Polymer 3/DNTF (0 1 1) are the largest one. Therefore, there is a conclusion that Polymer 3 can improve ductility of DNTF, and Polymer 1 and Polymer 2 can partly improve. The improvement of polymer binders in the ductility of DNTF decreases in the order: Polymer 3 > Polymer 2 > Polymer 1.

**Table 13:** The mechanical parameters of polymer/DNTF interfacial crystal.

System	Polymer 1/DNTF		Polymer 2/DNTF		Polymer 3/DNTF		DNTF
	(0 1 1)	(1 0 1)	(0 1 1)	(1 0 1)	(0 1 1)	(1 0 1)	
C_11_	2.2141	5.1373	3.0928	4.1046	6.1215	2.8775	12.6889
C_12_	1.2700	2.1021	0.9068	1.6871	1.6399	2.0411	3.9971
C_13_	1.0095	2.8320	1.3901	1.0004	1.6650	1.1242	1.1050
C_22_	3.9266	4.8189	1.3377	5.7476	0.8218	5.8535	10.8466
C_33_	1.2138	7.3288	4.9849	2.4391	0.9192	3.0028	9.6785
C_44_	1.3576	2.0329	1.4348	1.6087	0.9408	1.6451	2.8071
C_55_	0.5107	1.7170	0.9448	1.5080	0.4392	1.6477	2.6064
C_66_	1.4547	1.8851	1.1255	1.9422	0.8030	1.4729	8.1915
λ	0.2363	2.0049	0.8017	0.7245	1.1655	0.7341	2.0014
μ	1.1076	1.8784	1.1684	1.6863	0.7277	1.5886	4.5350
*E*/GPa	2.41	4.73	2.81	3.88	1.90	1.84	10.46
*K*/GPa	0.97	3.26	1.58	1.85	1.65	1.79	5.02
*G*/GPa	1.11	1.88	1.17	1.69	0.73	1.59	4.54
*γ*	0.09	0.26	0.20	0.15	0.31	0.16	0.15
*K/G*	0.87	1.73	1.35	1.09	2.26	1.13	1.11

#### Binder/CL-20 interfacial crystals

It can be found in Table 14 that the mechanical constants of three binder/CL-20 interfacial crystals all particularly decrease compared with those of pure CL-20, which implies three polymer binders all efficiently improve the isotropy of CL-20. Meanwhile, it is easy to see that the *E*, *K* and *G* values of polymer binder/CL-20 (0 1 1) (the first growth face, 56.4%) interfacial crystal are all lower than those of polymer binder/CL-20 (1 0 − 1) (the second growth face, 18.6%), which contribute to enhancing the ductility of system. Otherwise, the *E*, *K* and *G* values of Polymer 2/CL-20 and Polymer 3/CL-20 are smaller compared with those of Polymer 1/CL-20 when corresponding to same growth face (except *K* value of Polymer 2/CL-20 (1 0 − 1) interfacial crystal), which indicates that the modified P(BAMO/AMMO) can better improve the mechanical properties of CL-20. Moreover, contrasting the elastic constants of the modified P(BAMO/AMMO) combining with the first growth face of CL-20 (56.4%), it can be concluded that the ability of Polymer 3 reinforcing the ductility of CL-20 are superior to that of Polymer 2. In a conclusion, the ability of polymer binders decreasing the rigidity of CL-20 decrease in the order: Polymer 3 > Polymer 2 > Polymer 1. Otherwise, the *K*/*G* and *γ* values of Polymer 2/CL-20 and Polymer 3/CL-20 (0 1 1) interfacial crystals are all larger than those of CL-20 crystal, and the others are contrary. It is obvious that Polymer 2 and Polymer 3 can improve and partly increase the ductility of CL-20, respectively, but Polymer 1 fails, therefore, their ability is Polymer 2 > Polymer 3 > Polymer 1.

**Table 14:** The mechanical parameters of polymer/CL-20 interfacial crystal.

System	Polymer 1/CL-20		Polymer 2/CL-20		Polymer 3/CL-20		CL-20
	(0 1 1)	(1 0 − 1)	(0 1 1)	(1 0 − 1)	(0 1 1)	(1 0 − 1)	
C_11_	5.9783	3.3335	4.8436	2.7435	3.9254	3.8272	18.3688
C_12_	1.8597	2.4788	2.2585	2.4155	1.7400	2.6989	6.0061
C_13_	1.1368	1.9781	1.3475	1.9847	1.9235	1.3770	3.5932
C_22_	4.4738	10.3300	5.3651	5.3606	0.8391	7.7161	18.6105
C_33_	4.4527	7.4752	2.0624	8.5970	4.9251	4.7258	26.1583
C_44_	0.4587	1.8713	0.6785	1.1422	0.6525	1.0409	5.5171
C_55_	1.5460	2.5425	1.0361	0.6149	0.8773	1.6405	5.4845
C_66_	2.8784	4.0740	1.8533	2.4832	1.2196	2.7610	9.0163
λ	1.7128	1.3877	1.7118	2.7401	1.3969	1.7948	7.7006
μ	1.6277	2.8293	1.1893	1.4134	0.9165	1.8141	6.6726
*E*/GPa	4.09	6.59	3.08	3.76	2.39	4.53	16.92
*K*/GPa	2.80	3.27	2.50	3.68	2.01	3.00	12.15
*G*/GPa	1.63	2.83	1.19	1.41	0.92	1.81	6.67
*γ*	0.26	0.16	0.30	0.33	0.30	0.25	0.27
*K/G*	1.72	1.16	2.10	2.61	2.18	1.66	1.82

## Conclusion

Based on molecular dynamic method, two novel modified P(BAMO/AMMO) binders are designed, and their densities, glass transition temperatures (*T*_g_), volumetric coefficients of thermal expansion and mechanical properties are compared with P(BAMO/AMMO). Meanwhile, the effects of three polymer binders on mechanical properties of representative energetic materials (TNT, HMX, CL-20 and DNTF) of three generations are investigated, and the main results are as follow:

1. The densities of Polymer 1, Polymer 2 and Polymer 3 are respectively 1.229 g/cm^3^, 1.235 g/cm^3^ and 1.281 g/cm^3^, which shows introducing functional groups all increase the density of P(BAMO/AMMO). The *T*_g_ values of Polymer 1, Polymer 2 and Polymer 3 are respectively 247 ± 4 K, 240 ± 5 K and 181 ± 3 by calculating their *V*, *MSD* and *NBE* in different temperature, which suggests the the reducing effect of -NO_2_ group is better.
2. Except temperature is greater than *T*_g_ of Polymer 3 and less than *T*_g_ of Polymer 1, the Polymer 1 is much easiest to expand in whether rubbery state or glassy state, which may restrict its application. The introduced groups has few effect on diffusion coefficient of Polymer 1. The *E* and *G* values of modified P(BAMO/AMMO) are all lower than those of P(BAMO/ AMMO), despite it is contrary for the *K* value. It may be concluded that modified P(BAMO/AMMO) possess better mechanical properties .
3. The solubility parameters of three polymer binders approximate to each other, and their difference value (|Δ*δ*|) with four energetic materials is 8.08 (J/cm^3^)^1/2^ <|Δ*δ*|< 12.41 (J/cm^3^)^1/2^, there is a conclusion that three polymers are compatible with TNT, HMX and CL-20, but Polymer 2 and Polymer 3 may react with DNTF as same as Polymer 1.
4. Comparing the mechanical properties of interfacial crystal with corresponding crystal, it can be found that the effects of Polymer 2 and Polymer 3 reducing the rigidity of TNT are inferior to that of Polymer 1, but it is opposite for CL-20. Otherwise, there is only that the ability of Polymer 3 improving the rigidity of HMX and DNTF is superior to that of Polymer 1. Meanwhile, it is worth noting that the rigidity of Polymer 2-based systems are all higher than those of Polymer 3-based systems for four energetic material.
5. All polymers can improve ductility of four energetic materials (except Polymer 2 on TNT and Polymer 1 on CL-20). For TNT and HMX, the ability of Polymer 1 improving their ductility are superior to Polymer 2 and 3. However, it is contrary for DNTF and CL-20.
